# Evaluation of the Increase in Serum Calcium Levels After Unilateral Adrenalectomy

**DOI:** 10.7759/cureus.29132

**Published:** 2022-09-13

**Authors:** Mehmet Üstün, Korhan Tuncer

**Affiliations:** 1 Department of General Surgery, University of Health Sciences Tepecik Training and Research Hospital, İzmir, TUR; 2 Department of General Surgery, Izmir Bakircay University Cigli Training and Research Hospital, Izmir, TUR

**Keywords:** gfr, creatinine, adrenal insufficiency, hypercalcemia, adrenalectomy

## Abstract

Introduction

This study aims to investigate the prevalence and characteristics of patients with elevated serum calcium due to adrenal insufficiency after unilateral adrenalectomy.

Methods

The study included 76 patients who underwent unilateral adrenalectomy from January 2012 to November 2021 and did not have an additional etiologic factor for hypercalcemia, During the postoperative period, the highest calcium value in six months was taken into account as the postoperative value. Calcium values were corrected according to the albumin value.

Results

Of the 76 patients included in the study, serum calcium levels were higher in six patients (7.9%) after adrenalectomy. Unlike the others, a decrease in glomerular filtration rate (GFR) and an increase in serum creatinine values were detected in the postoperative period in this patient group. In this patient group, the corrected calcium level detected an average increase of 1.3 mg/dL.

Conclusion

After unilateral adrenalectomy, hypercalcemia may occur due to adrenal insufficiency. It should also be considered that there may be a decrease in GFR and increased creatinine in these patients.

## Introduction

Hypercalcemia and its various symptoms are an important reason for hospitalization, considering the diversity of its underlying causes. Although its incidence in the general population is 1/1000, it constitutes 0.6% of all acute medical admissions [1]. The symptoms may vary depending on the serum calcium level. Most commonly, it’s detected in laboratory tests without any symptoms. Depending on the severity of hypercalcemia, nausea, vomiting, ventricular fibrillation, and associated cardiac arrest, it can result in QT prolongation, confusion, and coma [1]. The common causes of hypercalcemia are primary hyperparathyroidism (PHP), cancer, sarcoidosis, and use of drugs such as thiazide diuretics, calcitriol, lithium, vitamin A and D intoxications, milk-alkali syndrome, familial hypocalciuric hypercalcemia (FHH), and untreated Addison's disease (primary adrenal insufficiency). Because symptoms of other hormone deficiencies are more prominent in this scenario, hypercalcemia secondary to adrenal insufficiency is frequently overlooked [2]. Some studies in the literature report that elevated serum calcium is more common in adrenal insufficiency than previously thought [3,4].

The purpose of this study is to look at the prevalence and characteristics of patients who had hypercalcemia after unilateral adrenalectomy due to adrenal insufficiency.

## Materials and methods

Patients who underwent unilateral adrenalectomy between January 2012 and November 2021 and who did not have additional etiologic factors for hypercalcemia were enrolled. All patients underwent a thorough examination by anesthesiologists to be eligible for elective surgery, and none of them required emergency interventions. The electrolyte levels of patients with electrolyte imbalance were corrected in the preoperative period, and none of the patients had problems with acid-base balance. In patients with more than one laboratory examination in the preoperative period, the highest serum calcium levels were accepted as the baseline value.

In patients with low albumin levels, calcium levels were corrected using the formula "Corrected calcium (mg/dL) = Total calcium (mg/dL) + 0.8 x (4.0 - albumin (g/dL)). During the postoperative period, the highest calcium value in the six months was taken into account as the postoperative value.

The increase in serum calcium levels over 1 mg/dL during this period was considered significant [5].

Hospital records were reviewed; demographics, preoperative clinical diagnosis, histopathological results, preoperative and postoperative calcium, phosphorus, albumin, corrected calcium levels, and glomerular filtration rates (GFR) (calculated by the Modification of Diet in Renal Disease (MDRD) formula) of patients with increased serum calcium levels after adrenalectomy were noted.

The study was approved by the Ethics Committee of the University of Health Sciences Tepecik Training and Research Hospital (Approval Number: 2022/05-07) and is in adherence to the Helsinki Declaration.

Statistical analysis

Statistical analyses were done with IBM's Statistical Package for Social Sciences (SPSS) software, version 25.0. The number of units (n), percent (%), mean ± standard deviation (SD), and median (Q1-Q3) values were given as descriptive statistics. Pearson Chi-square tests were used to evaluate categorical variables. The normality of the distribution of continuous variables was evaluated by the Shapiro-Wilk normality test and quantile-quantile (Q-Q) graphs. In comparison between the continuous variables of the two groups, the independent sample t test was used for variables with a normal distribution and the Mann-Whitney U test for variables that did not fit the normal distribution. A p-value of 0.05 was considered statistically significant.

Estimation of the glomerular filtration rate

The GFR was estimated by using the MDRD formula [6]. In these equations, GFR and creatinine clearance (Ccr) are expressed as mL per minute per 1.73 m2, and serum creatinine levels (Scr) are expressed as mg/dL. The value found for black races should be multiplied by 1.21.

GFR = 186 x ((Scr) -1.154) x ((Age) -0.203) x (0.742 if female)

## Results

Seventy-six patients were enrolled, out of which 14 (18.4%) were male and 62 (81.6%) were female. The mean age was calculated to be 52.8±12.5 years. Forty-six (60.5%) patients had clinically nonfunctional adrenal mass. Pheochromocytoma was in 20 (26.3%) patients, Cushing's disease in 5 (6.6%) patients, aldosteronoma in 1 (1.3%) patient, adrenocortical carcinoma in 2 (2.6%) patients, and cancer metastasis in 2 (2.6%) patients. Clinical and histopathological diagnoses are given in Table 1.

**Table 1:** Clinical and histopathological diagnoses

	All patients	Serum calcium increased <1	Serum calcium increased >1
	n=76	n=70	n=6
Clinical diagnosis, n (%)			
Adrenal adenoma	24 (31,6)	23 (32,9)	1 (16,7)
Adrenal mass	25 (32,9)	23 (32,9)	2 (33,3)
Pheochromocytoma	20 (26,3)	18 (25,7)	2 (33,3)
Cushing's syndrome	5 (6,6)	4 (5,7)	1 (16,7)
Aldosteronoma	1 (1,3)	1 (1,4)	0
Adrenal cyst	1 (1,3)	1 (1,4)	0
Pathologic diagnosis, n (%)			
Adrenal cortical adenoma	31 (40,8)	28 (40)	3 (50)
Pheochromocytoma	14 (18,4)	13 (18,6)	1 (16,7)
Myelolipoma	7 (9,2)	6 (8,6)	1 (16,7)
Adrenal cortical hyperplasia	4 (5,3)	4 (5,7)	0
Endothelial cyst	4 (5,3)	4 (5,7)	0
Adrenal adenoma	2 (2,6)	2 (2,9)	0
Adrenal pseudocyst	2 (2,6)	2 (2,9)	0
Adrenocortical carcinoma, oncocytic variant	2 (2,6)	2 (2,9)	0
Lipoma	2 (2,6)	2 (2,9)	0
Benign corticomedullary mixed tumor	1 (1,3)	1 (1,4)	0
Benign adrenal cyst	1 (1,3)	1 (1,4)	0
Bleeding and degenerative changes	1 (1,3)	1 (1,4)	0
Cystic vascular hamartoma	1 (1,3)	1 (1,4)	0
Colon adenocarcinoma metastasis	1 (1,3)	0	1 (16,7)
Corticomedullary adenoma	1 (1,3)	1 (1,4)	0
Xanthogranulomatous adrenalitis	1 (1,3)	1 (1,4)	0
Malignant epithelial tumor lung metastasis	1 (1,3)	1 (1,4)	0

Severe symptoms of hypercalcemia did not occur in any patient. Six patients (7.9%) had a serum calcium level increase higher than 1 mg/dL after adrenalectomy. An average increase of 1.3 mg/dL was detected in the corrected calcium level. These patients did not differ from the whole group in terms of age, sex distribution, and etiology (Table 2). One (16.7%) of these patients was male, while five (83.3%) were female. The mean age of the patients was 55.2 years. Two (33.3%) patients had non-functional adrenal masses; two (33.3%) had pheochromocytoma; one (16.7%) had Cushing's disease, and one (16.7%) had colonic adenocarcinoma metastasis. Unlike the others, a decrease in GFR and an increase in serum creatinine values was detected during the postoperative period in this patient group. Demographics and clinical data for this patient group are given in Table 2.

**Table 2:** Demographics and clinical data of this patient group *Fisher's exact test was used.

	All patients	Serum calcium increased <1	Serum calcium increased >1	
	n=76	n=70	n=6	p-value
Age, mean±SD	52,8±12,5	52,6±12,7	55,2±8,9	0,626
Gender, n (%)				1,000*
Male	14 (18,4)	13 (18,6)	1 (16,7)	
Female	62 (81,6)	57 (81,4)	5 (83,3)	
Preoperative results				
Serum calcium (mg/dl), mean±SD	9,8±0,4	9,8±0,4	9,6±0,2	0,201
Serum phosphorus (mg/dl), mean±SD	3,6±0,5	3,6±0,5	3,3±0,5	0,154
Serum albumin (g/dl), mean±SD	4,3±0,3	4,3±0,3	4±0,3	0,010
Serum creatine, median (Q1-Q3)	0,8 (0,7-0,9)	0,8 (0,7-0,9)	0,9 (0,7-1,1)	0,653
GFR-MDRD, mean±SD	77,7±17,6	78,1±17,9	72,6±14,3	0,465
Corrected calcium (mg/dL), mean±SD	9,6±0,4	9,6±0,4	9,6±0,2	0,965
Postoperative results				
Serum calcium (mg/dl), mean±SD	9,4±0,8	9,3±0,7	10,7±0,8	<0,001
Serum phosphorus (mg/dl), mean±SD	3,8±0,6	3,7±0,6	3,8±0,8	0,737
Serum albumin (g/dl), mean±SD	3,9±0,5	3,9±0,5	3,8±0,4	0,528
Serum creatine, median (Q1-Q3)	0,8 (0,7-1)	0,8 (0,7-0,9)	1,2 (0,9-1,5)	0,009
GFR-MDRD, mean±SD	77,5±20,1	79,5±19	54,8±19,7	0,003
Corrected calcium (mg/dL), mean±SD	9,5±0,7	9,4±0,5	10,9±0,5	<0,001

## Discussion

Hypercalcemia because of adrenal insufficiency due to various reasons has been reported in various studies [3,7,8]. Unilateral adrenalectomy is among the causes of adrenal insufficiency [9]. There is only one study reporting the incidence of hypercalcemia due to adrenal insufficiency after adrenalectomy. Kim et al. reported the incidence of hypercalcemia due to unilateral adrenalectomy as 7.5% in a series of 239 patients in 2022 [5].

Our series reveals a similar incidence, and more importantly, all patients in our study had asymptomatic hypercalcemia. It is very difficult to determine the diagnosis of adrenal insufficiency after adrenalectomy in patients with asymptomatic hypercalcemia. Kim et al. defined a 1 mg/dL increase in calcium levels as a significant change in serum calcium levels [5]. We also considered an increase of 1 mg/dL as significant in our study. While the mean preoperative calcium level was 9.6, the mean calcium level was found to be 10.9 in the group with significant calcium elevation in the postoperative period.

Hypercalcemia secondary to unilateral adrenalectomy is an unexpected finding; however, both Kim et al. [5] and our series revealed a similar incidence. There are various theories about the mechanism of hypercalcemia due to adrenal insufficiency. Kim et al. attributed hypercalcemia after adrenalectomy to three different mechanisms. One of these may be decreased inhibition of the 1-alpha-hydroxylase enzyme due to decreased prednisolone levels. Another mechanism that is thought to be effective in the etiology may be the decrease in glomerular filtration rate due to hypovolemia caused by adrenal insufficiency. Decreased stanniocalcin secretion from the adrenal gland may cause hypercalcemia by inducing calcium secretion from the blood skeletal system into the blood circulation [5].

Kim et al. reported a relationship between lower than normal contralateral adrenal volume and the development of hypercalcemia after adrenalectomy [5]. Our study cannot give data on this hypothesis as adrenal gland volume was not measured. On the other hand, our study revealed an increase in postoperative creatinine and a significant decrease in GFR in patients with significantly higher calcium levels despite similar preoperative creatinine levels and GFR between the two groups.

Our findings support their second hypothesis, as hypovolemia is caused by adrenal insufficiency, though its mechanism has not been fully elucidated. Current data in the literature also indirectly support our findings [5]. Kim et al. reported that patients with primary aldosteronism after unilateral adrenalectomy had a significant tendency to decrease GFR in the first month compared to others [10]. Park et al. reported a decrease in GFR and an increase in creatinine in patients who developed hyperkalemia after adrenalectomy [11]. Primary aldosteronism was not found in any of the patients who developed significant hypercalcemia in our study. But, to our knowledge, hypercalcemia associated with decreased GFR after unilateral adrenalectomy is reported for the first time.

Limitations

Our study has some limitations. The study was retrospective, and data were collected according to standard protocols whenever possible. The timing of laboratory tests differed between patients. Although the highest calcium level in the six-month postoperative period was evaluated, it may not have been measured because the patients were asymptomatic. Patients' comorbidities and medications were not taken into account. It was not calculated whether the adrenal mass was functional or not. The limited number of studies in the literature reduces comparability.

## Conclusions

In our study, hypercalcemia was detected in 7.9% of patients after unilateral adrenalectomy. This may be due to adrenal insufficiency. In patients undergoing unilateral adrenalectomy, hypercalcemia and the associated decrease in GFR will be investigated further. The authors should consider including patients undergoing unilateral adrenalectomy for other etiologies such as primary or metastatic malignancies. Prospective studies with planned, larger investigations to uncover etiopathogenesis are required. 
